# Concomitant acute interstitial nephritis and autoimmune hemolytic anemia induced by omeprazole

**DOI:** 10.1002/ccr3.2661

**Published:** 2020-01-15

**Authors:** Neetu Sharma, Randy Ip, Tarik Hadid

**Affiliations:** ^1^ School of Medicine Oakland University Rochester MI USA; ^2^ Graduate Medical Education Department of Internal Medicine Ascension Macomb‐Oakland Hospital Warren MI USA; ^3^ School of Medicine Wayne State University Detroit MI USA

**Keywords:** acute interstitial nephritis, hemolytic anemia, omeprazole, proton pump inhibitors

## Abstract

Proton pump inhibitors (PPI)—induced acute interstitial nephritis and autoimmune hemolytic anemia can occur concomitantly, which should prompt discontinuation of PPI. PPI should wisely be prescribed and discontinued when no longer needed.

## Graphical abstract

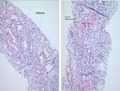

## 1 INTRODUCTION

Proton pump inhibitors (PPI) are commonly prescribed worldwide. PPI are rarely associated with acute interstitial nephritis (AIN) and autoimmune hemolytic anemia (AIHA). We report a unique patient who simultaneously developed AIN and AIHA with complete recovery with PPI discontinuation. PPI should wisely be prescribed and discontinued when no longer needed.

Proton pump inhibitors (PPI) are widely used for the prevention and treatment of peptic ulcer disease (PUD), erosive and nonerosive esophagitis and Zollinger‐Ellison syndrome.^1^ Omeprazole is the prototype of PPI. In the United States, multiple PPI such as lansoprazole, and esomeprazole and omeprazole magnesium are available over the counter. PPI inhibit the H+/K+‐ATPase pumps on the surface of the parietal cells, the final step in the hydrochloric acid (HCL) secretary process, resulting in marked decrease in HCL production. PPI are effective and safe with only limited side effects such as increased risk for osteoporosis, hip fractures, *Salmonella*, *Campylobacter jejuni*, and *Clostridium difficile* infections, vitamin B12 deficiency, and electrolytes imbalance.^2^ Serious and life‐threatening adverse effects of PPI such as Steven‐Johnson syndrome and toxic epidermal necrolysis are extremely uncommon and only reported sporadically in the literature.^3^

Renal and hematologic toxicities of PPI are exceedingly rare. We report a patient who concomitantly developed autoimmune hemolytic anemia (AIHA) and acute interstitial nephritis (AIN) along with acute tubular necrosis (ATN) due to use of PPI.

## 2 CASE REPORT

A 43‐year‐old previously healthy woman presented with generalized weakness and fatigue. She reported no shortness of breath, palpitation, chest pain, or clinical symptoms of bleeding. Physical examination was unremarkable except for conjunctival pallor. Complete blood counts revealed hemoglobin of 5.7 gm/dL, white blood cells 3200/µL, platelet count 295 000/µL, mean corpuscular volume 55.7 fL, ferritin 3 mg/mL, and iron saturation 2.5%. She received 2 units of packed red blood cells, intravenous famotidine, and a short course of intravenous ferrous gluconate. She was subsequently discharged on oral ferrous sulfate and omeprazole for empiric treatment of PUD and recommended for outpatient endoscopic examination. Her hemoglobin at the day of discharge was 8.5 gm/dL.

**Figure 1:**
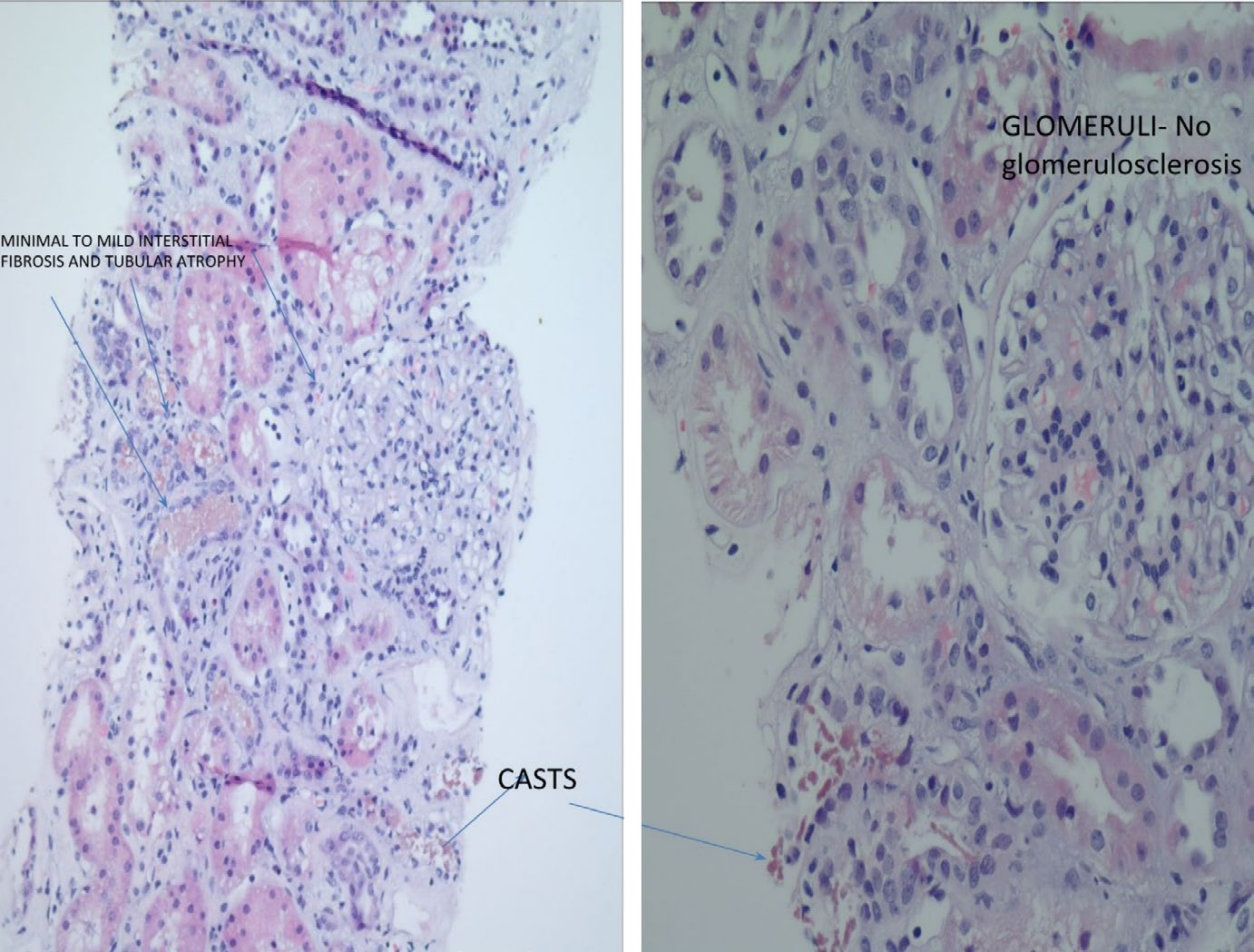
A myoglobin immunostain on kidney biopsy fails to stain granular casts, ruling out myoglobin nephropathy. There is mild interstitial fibrosis and minimal tubular atrophy (left) with normal glomeruli (right)

**Figure 2:**
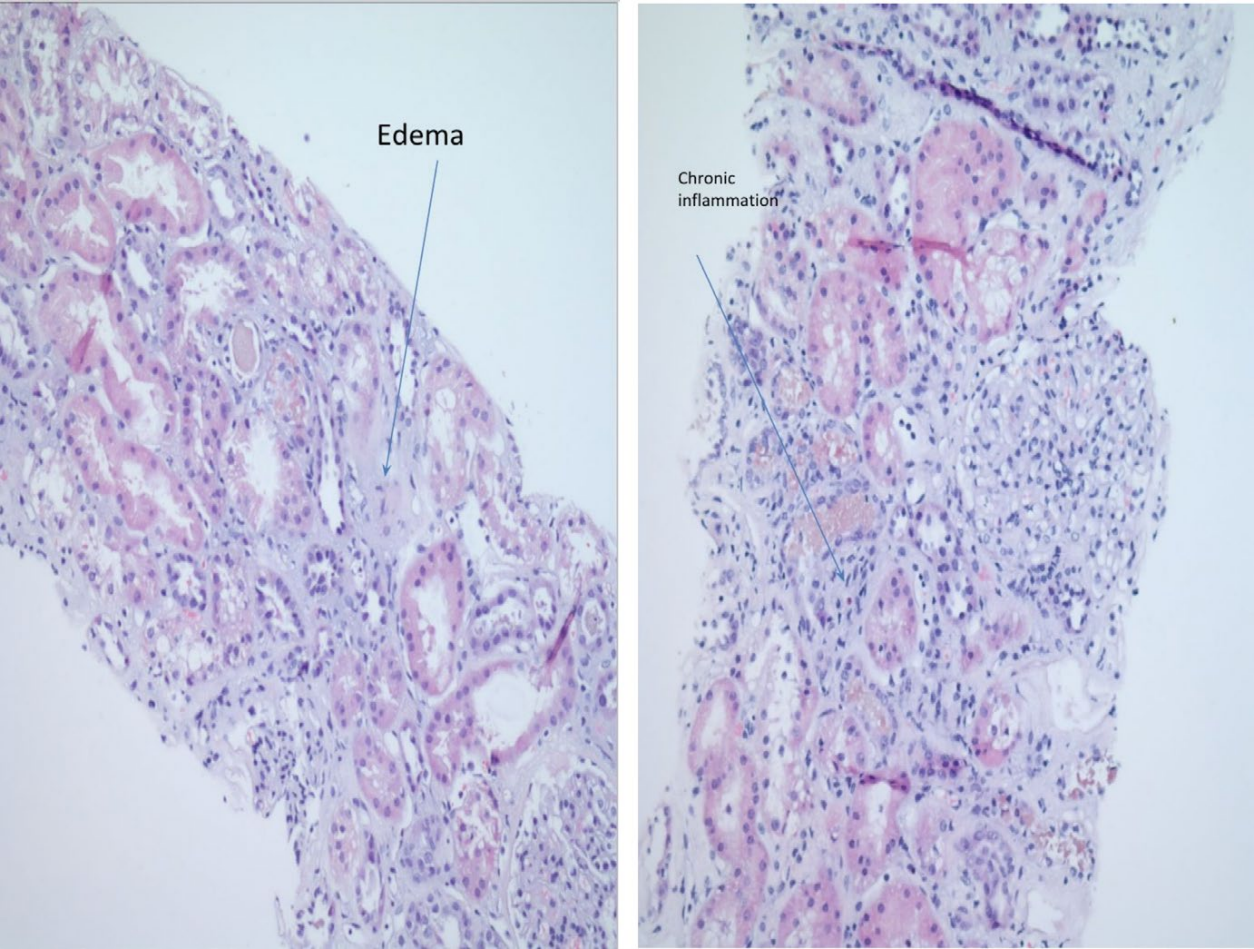
The renal interstitium contains focally moderate lymphocytic and eosinophilic cellular infiltrates with associated edema and chronic inflammation (arrows)

The patient returned to the emergency department 9 days later with worsening weakness, intractable nausea and vomiting and decreased oral intake for a few days. She denies consumption of nonsteroidal anti‐inflammatory drugs. Her medications were limited to ferrous sulfate, omeprazole, and ergocalciferol. Upon presentation, she was found to have severe anemia with hemoglobin of 7.4 gm/dL which subsequently further declined to 6.2 gm/dL. She was also found to have acute kidney injury with creatinine of 5.17 mg/dL, which further progressed to peak at 15.09 mg/dL. Laboratory studies revealed improving iron parameters with normal vitamin B12 and folic acid levels. Due to concern about hemolysis, lactic dehydrogenase was checked and found to be elevated at 1155 IU/L, which then further progressed to peak at 1769 IU/L. Haptoglobin was <10 mg/dL, and plasma free hemoglobin was detected at 9 mg/dL. Coomb's test was negative, but super‐Coombs was positive. Paroxysmal nocturnal hemoglobinuria panel and glucose‐6‐phosphate dehydrogenase levels were normal. Peripheral blood smear showed no schistocytes making the diagnosis of microangiopathic hemolytic anemia unlikely. Antinuclear antibody was weakly positive with a titer of 1:80. Anti‐smith antibody, antideoxynucleic acid antibody, C3, C4, and serum immunofixation studies were all unremarkable. Cytoplasmic‐neutrophil cytoplasmic antibodies (C‐ANCA) was weakly positive with a titer of 1:40. Serologic testing for human immunodeficiency virus and hepatitis B and C were negative. The patient was diagnosed with autoimmune hemolytic anemia (AIHA) and was initiated on intravenous methylprednisolone 1000 mg daily for 3 days along with plasmapheresis. Bone marrow aspiration and biopsy were performed to assess for possible lymphoproliferative disorder, which was negative. Renal biopsy was performed which revealed acute tubular injury with necrosis with eosinophilic granular casts, patchy, mild‐focally moderate lymphocytic interstitial infiltrate and interstitial edema. No global glomerulosclerosis with only minimal to mild interstitial fibrosis, tubular atrophy, and mild arteriosclerosis. Myoglobin immunostain and immunofluorescence of light chains were negative without evidence of active vasculitis or thrombotic microangiopathy (Figures 1 and 2). These findings are consistent with concomitant diagnosis of AIN and ATN most likely induced by omeprazole.

The patient was continued on 3‐month tapered course of prednisone. Omeprazole was permanently discontinued. Two weeks later, kidney function improved and hemoglobin stabilized. Outpatient follow‐up confirmed complete hematologic and renal recovery.

## 3 DISCUSSION

PPI have been widely prescribed for the management of gastroesophageal reflux symptoms since their discovery in 1980s. Omeprazole was introduced as the first effective PPI in 1989.^2^ Most studies have supported a mild side effect profile ranging from headaches and dizziness to abdominal pain and diarrhea. Hemolytic anemia and AIN are considered rare side effects of PPI with only few case reports described patients with PPI‐induced hemolytic anemia and a few others reported patients with PPI‐induced AIN.^2^, ^4^

Drug‐induced autoimmune hemolytic anemia is commonly associated with the use of antibiotics. Drug‐induced AIHA is believed to be significantly underestimated likely due to underdiagnosis. There are two types of antibodies that have been associated with drug‐induced AIHA; drug‐independent antibody that can be detected in vitro without the addition of the drug and drug‐dependent antibody that reacts in vitro only in the presence of the drug.^5^ Interestingly, it remains unclear why and how certain drugs can induce RBC autoantibody formation without necessarily causing a hemolytic anemia.^6^ However, there is a universally accepted mechanism through which drug‐dependent hemolytic anemia develops. Certain drugs can bind to RBC surface proteins covalently, and at high enough concentrations, the RBC will be coated with the drug. While this is typically a benign process, some patients may develop IgG autoantibodies that can bind to the drug‐RBC protein surfaces leading to complexes that are targeted by the immune system for destruction and ultimately hemolysis.^5^ This theory explains why these patients usually develop Coombs positive AIHA. Our patient had AIHA with positive super‐Coombs test and responded well to steroids, which strongly suggests autoimmune mechanism of hemolysis most likely triggered by the use of omeprazole.

PPI‐induced AIN is rarely reported in literature.^7^, ^8^ Of the reported cases, the majority were linked to the use of omeprazole possibly due to its longer availability for clinical use and its inherent immunogenicity.^2^ The largest series to date was reported by Geevasinga et al who described 18 cases of biopsy‐proven PPI‐induced AIN diagnosed over 10 years in two large Australian hospitals, which represent 64% of all biopsy‐proven AIN cases within this period. Among these patients, 11 were induced by omeprazole, 3 by pantoprazole, 3 by esmeprazole, and 1 by rabeprazole. AIN developed at a mean of 11 weeks following initiation of PPI.^4^ The presentation of PPI‐induced AIN is variable. While 10% of reported cases in literature have classical hypersensitivity triad of fever, rash, and eosinophilia, the majority of patients, however, had nonspecific complaints including weakness, fatigue, nausea, and vomiting as in our patient.^9^ Diagnosis of PPI‐induced AIN is suspected based on history but should be confirmed with renal biopsy given the variability of clinical presentation. Renal biopsy typically shows interstitial infiltrates with or without tubular injury and tubulitis. Cellular infiltrates in the interstitium consist mostly of eosinophils and lymphocytes. Glomeruli in most cases are normal. Management of PPI‐induced AIN and hemolytic anemia relies mainly on discontinuation of the offending drug. However, early initiation of steroids may hasten renal recovery and improve hemolysis.^10^

Although PPI‐induced AIHA and AIN were individually reported in the literature,^2^, ^4^, ^7^, ^8^ to our knowledge, this is the first case to report these two entities occurring concomitantly. Fortunately, the offending drug was promptly discontinued, and the patient achieved complete recovery.

## 4 CONCLUSION

While PPI are often safe, they can cause serious complications such as AIN and AIHA. Rarely, more than one PPI‐induced complications can simultaneously occur. Therefore, PPI should judiciously be prescribed. Early diagnosis of PPI‐induced adverse effects is essential to prompt discontinuation of PPI and initiation of supportive therapy to improve clinical outcomes.

## CONFLICT OF INTEREST

All authors disclose that they have no conflict of interests related to this manuscript. All authors contributed to this manuscript.

## AUTHOR CONTRIBUTIONS

Neetu Sharma, MD: extensively reconstructed, edited, and reviewed the details of this manuscript. Randy Ip, MD: wrote the initial manuscript, which was then further edited and reviewed by the other authors. Tarik Hadid, MD, MPH, MS: provided the final review and edit of this manuscript and formatted it in its final form.

## CONSENT

The patient has provided written informed consent for publication.
